# Knowledge and Self-Reported Practice of Insulin Injection Device Disposal among Diabetes Patients in Gondar Town, Ethiopia: A Cross-Sectional Study

**DOI:** 10.1155/2016/1897517

**Published:** 2016-09-25

**Authors:** Abebe Basazn Mekuria, Begashaw Melaku Gebresillassie, Daniel Asfaw Erku, Kaleab Taye Haile, Eshetie Melese Birru

**Affiliations:** ^1^Department of Pharmacology, College of Medicine and Health Sciences, University of Gondar, Chechela Street, Lideta Subcity Kebele 16, Gondar, Ethiopia; ^2^Department of Clinical Pharmacy, School of Pharmacy, University of Gondar, Chechela Street, Lideta Subcity Kebele 16, Gondar, Ethiopia; ^3^Department of Pharmaceutics, School of Pharmacy, University of Gondar, Chechela Street, Lideta Subcity Kebele 16, Gondar, Ethiopia

## Abstract

*Background.* Incorrect sharp disposal practices may expose the public to needle-stick injuries. The present study aimed at assessing the knowledge and practice of diabetic patients towards insulin injection device disposal in Gondar town, Ethiopia.* Methods.* A cross-sectional study was employed on insulin requiring diabetes patients who visited the diabetes clinic at Gondar University Referral Hospital (GURH) from February 1 to March 28, 2016. Frequencies, percentages, and ANOVA (analysis of variance) and Student's* t*-test were used to analyze variables.* Results.* About half of the participants (49.5%) had poor knowledge towards safe insulin injection waste disposal. More than two-thirds (80.7%) of respondents had poor practice and 64.3% of respondents did not put insulin needle and lancets into the household garbage. 31% of respondents threw sharps on street when they travel outside. Respondents living in urban areas had a higher mean of knowledge and practice score than those who live in rural area.* Conclusions.* This study revealed that knowledge and practice of diabetic patients were low towards safe insulin injection waste disposal in study area. Healthcare providers should also be aware of safe disposing system and counsel patients on appropriate disposal of used syringes.

## 1. Introduction

Diabetes mellitus is a metabolic disorder characterized by chronic hyperglycemia [1]. It is a common chronic disease its management needs regular blood tests and insulin injections [2]. The management mostly takes place by self-administration of insulin at home. Various kinds of medical instruments, like insulin pens, needles, and syringes, as parts of self-care, are used [3]. Sharps have been defined by World Health Organization as “items that could cause cuts or puncture wounds, including needles, hypodermic needles, scalpel, and other blades, knives, infusion sets, saws, broken glass, and nail” [4].

Proper way of disposing sharps is one of the important, but often neglected, components of proper injection techniques [5]. In some countries, reuse of nonsterile insulin injection tools was a common malpractice in society [6]. Incorrect sharp disposal practices among diabetes patients and members of their family lead to needle-stick injuries among rag pickers, domestic waste handlers, and the community [7]. Injuries from contaminated needles and sharps present a concern in society today since they may increase risk for the transmission of blood-borne pathogens, such as human immunodeficiency virus, hepatitis B, and hepatitis C [6–8]. The barriers to safe disposal of sharps by diabetes patients include lack of information about how and where to dispose, lack of proper advice from healthcare practitioners, wrong perception on sharp disposal, and self-administration of insulin by diabetic patients [9, 10].

Study done in different countries showed that proportion of various kinds of sharps thrown into the household bin varied from 46.9% to 67.6%. Only in less than 10% of cases specific containers were used to dispose insulin injection waste [6]. Other studies done in developed countries have also showed improper sharp disposal practices among diabetes patients to be as high as 80–90% [10, 11]. A study from Pakistan showed that more than 90% patients discarded injection devices into the household bin [12]. However, there is a paucity of data regarding the knowledge and practice of Ethiopian diabetes patients towards insulin injection device disposal. The present study aimed at assessing the knowledge and practice of diabetic patients towards insulin injection device disposal in Gondar town, Ethiopia.

## 2. Materials and Methods

### 2.1. Study Design and Setting

A cross-sectional study was employed on insulin requiring diabetes patients who visited the diabetes clinic at Gondar University Referral Hospital (GURH) from February to March 2016. GURH is located in Gondar town, Northwest Ethiopia, 738 km away from Addis Ababa (the capital city of Ethiopia). It is one of the oldest and pioneering referral hospitals in Ethiopia with a range of specialists including a diabetes illness follow-up care clinic, which currently provides free service for more than 5,000 diabetic patients annually on outpatient level.

### 2.2. Population and Sampling

All insulin requiring type 1 and type 2 diabetes mellitus patients who visited the diabetes clinic of GURH from February 1 to March 28, 2016, for control of blood sugar and medication refill were included in the study. We chose the 2-month follow-up period for data collection to avoid duplication of the cases as patients return to the clinic every 2 months. About 720 patients visited the clinic in the 2-month period. Every second person encountered and who meet the inclusion criteria was included. Thus, a total of 210 study subjects were selected through a systematic sampling procedure (study subjects were selected from the source population according to a random starting point and a fixed periodic interval).

### 2.3. Data Collection and Management

Data collection was performed by three well trained nurses through interviewer-administered questionnaires. The questionnaire was designed after a thorough literature review of the relevant available studies [9, 13]. The content validity of the questionnaire was confirmed by a team of experts including two endocrinologists and a senior nurse. The questionnaire was first written in English and translated to local language (Amharic) which is then translated back to English in order to ensure that the translated version gives the proper meaning. The questionnaire was pretested on 15 participants prior to the gross data collection, which were not included in the final analysis, and relevant modifications were instituted prior to commencement of actual data collection. The final questionnaire consisted of 46 items which were divided into two parts. Part one assesses the sociodemographic and treatment related characteristics of respondents including age, sex, marital status, educational level, age at diagnosis, duration of insulin use, and family history of DM. Part two includes questions regarding the knowledge and practice towards insulin injection device disposal. Patients' knowledge was assessed using 12 dichotomous (true/false) questions about sharp use, reuse, and sharp waste disposal. The statements had only one “correct” option and the respondents got 1 score for correct answer and 0 score for incorrect answer. The obtained score of knowledge was then classified using mean and standard deviation as “Good Knowledge” (score of 8–12), “Moderate Knowledge” (score of 4–7), and “Poor Knowledge” (score of 0–3). Patients' practices were assessed using 12 questions on using household garbage bags for disposal, frequency of needle reuse, and information seeking of patients regarding insulin ejection device disposal, which were answered as either “yes” or “no.” A score of 1–8 was considered “Negative Practice” while a score of 9–12 was considered “Positive Practice.”

### 2.4. Statistical Analysis

The final data collection tool was ensured for completeness, and responses were entered into and analyzed by the Statistical Package for the Social Sciences (SPSS) software version 21.0 for Windows. Frequencies and percentages were used to express different variables. One-way ANOVA and Student's *t*-test were used to compare groups. All statistical tests were performed using 0.05 as the level of significance.

### 2.5. Ethical Considerations

This study was approved by the ethical committee of University of Gondar, School of Pharmacy. Informed consent was also obtained from each participant before conducting this study. Participants' information obtained was kept confidential.

## 3. Results

Of the 210 respondents, 56.2% were males and 39.5% was in the age group of 20–39 years with the mean ± SD of 43.9 ± 15.8 years. Majority of the respondents were Orthodox Christians (62.4%) and urban residents (53.3%). Among the respondents, 170 (80.9%) were diabetes type 1 patients and 125 (59.5%) had family history of DM. More than half of the respondents (60.5%) use insulin for more than 5 years. Other patients' sociodemographic and clinical characteristics are shown in Tables 1 and 2, respectively.

The mean knowledge score was 6.7, which fall within our definition of “Moderate Knowledge.” About half of the participants (49.5%) had poor knowledge, 29.5% had medium level of knowledge, and the remaining 21% of respondents had good knowledge about insulin injection device disposal. Percentages of correct and incorrect answers to questions on knowledge towards insulin injection device disposal are shown in Table 3. About two thirds of respondents (63.3%) did not know how to dispose lancets after use and 56.7% respondents said that insulin injection device cannot be recycled like plastics. More than two thirds of respondents (72.5%) cited doctors as the primary source of acquiring information about insulin injection device disposal.

Analysis showed that more than two thirds (80.7%) of respondents had poor practice and most of respondents (64.3%) did not put insulin needle and lancets into the household garbage bags. Similarly, about 31% of respondents threw sharps on street when they travel outside. Furthermore, majority of the respondents (79%) did not ask their healthcare providers about disposal of insulin injection syringes. Interestingly, 57.1% of respondents bend needle and sharps after usage (Table 4).

One-way ANOVA with post hoc test showed significant difference in knowledge and practice of respondents towards insulin injection device disposal among rural and urban residents where respondents living in urban areas had a higher mean knowledge and practice score (mean: 6.81 (0.86), *p* value 0.003 and mean: 8.88 (0.54), *p* value 0.001, resp.) than those who live in rural areas (mean: 4.89 (0.77) and 5.45 (0.61), resp.). The overall knowledge and practice score was higher in those who join college or university (mean: 7.89 (0.73), *p* value 0.020) than in those who are illiterate (mean: 5.23 (0.85)). Respondents with a family history of DM patients had a significantly higher knowledge score (mean: 8.61 (0.99), *p* value 0.026) than those who did not have a family history of DM (mean: 6.34 (0.97)). Type of DM and duration of insulin use also affect the overall knowledge and practice where respondents with type 1 DM (mean: 8.23 (0.77), *p* value 0.000 and mean: 9.98 (0.68), *p* value 0.005, resp.) and who use insulin for more than 5 years (mean: 9.12 (0.99), *p* value 0.006 and mean: 9.13 (0.14), *p* value 0.000, resp.) had a higher knowledge and practice mean scores than those who had type 2 DM (mean: 6.09 (0.81) and 5.45 (0.60), resp.) and use insulin for less than 1 year (mean: 6.43 (0.83) and 5.78 (0.66), resp.). The knowledge and practice of respondents who visit their physician and get information more than two times (mean: 8.23 (0.91), *p* value 0.031 and mean: 9.78 (0.71), *p* value 0.001 resp.) were higher than those who visit their physicians only when they get sick and do not have information (mean: 5.76 (0.76) and 4.12 (0.60), resp.) (Table 5).

## 4. Discussion

This study assessed the knowledge and practice of diabetic patients toward insulin injection device disposal in Gondar town, which is located Northwest Ethiopia. The disposal of insulin injection is ignored by most health practitioners and governments. This exposes patients to dispose injection syringes everywhere. Syringe disposal without effective waste disposal system opens a new portal of transmission of blood-borne pathogen from patients to general community [14–20]. Disposal of used injection equipment at open place in rural areas could increase the risk of transmission to children, who play with syringes and can get pricked [21], and this correlated with transmission of blood-borne infection like HIV, HBV, and HCV to community [22–25].

In the present study, half of the participants (49.5%) had poor knowledge, 29.5% had medium level of knowledge, and the remaining 21% of respondents had good knowledge about insulin injection device disposal. This finding was of comparably higher values than the study conducted in Delhi, India, where those who had poor knowledge were 31 (10.3%), was of lower values in those who had moderate knowledge (65.9%), and was of similar values in those who had high level of knowledge (23.8%) [26]. In this survey, about two thirds of respondents (63.3%) did not know how to dispose lancets after use. This finding is of similar value to that of a study conducted in UK (64.9%) [27] but of a lower value than that of a study done in India (96.4%) [26]. 

Regarding the source of information, in this study 72.5% cited doctors, 18.1% cited nurses, and none of them cited pharmacist as the primary source of acquiring information about insulin injection device disposal. But a study done in Virginia in 2010, pharmacists and nurses, 25.0% and 40.0%, were cited as the primary source of acquiring information, respectively [28]. This might have been the reason of the results obtained in Ethiopia, where patients traditionally believe physicians, rather than pharmacists or nurses, are the source of health information. The current study revealed that 80.7% of respondents had poor practice of insulin device disposal. This finding was much higher compared to other studies (31.0%) [26]. This might be due to the fact that our country has low social media coverage in health sector and suffers from absence of public health education and absence of safe disposing system, and less concern is given by the government to proper waste disposal.

In the present study, about 40% respondents had disposed of insulin injection device in the toilet. However, the UK Diabetes Guidelines recommended the use of opaque hard plastic containers for disposing sharp waste [29]. This finding suggested that in the study area there is malpractice of disposing insulin injection waste. On the other hand, one third of patients 36.2% disposed of insulin injection device directly into household garbage bin. This finding is similar to another study done in Stafford, where 35.1% of patients disposed of their lancets and syringes into household garbage bin [27]. However, it was of lower value than those of a study done in Virginian 50% and France 49.9% [28, 30]. 72.4% of diabetic patients in our study never recap insulin syringe during disposal. This finding was in agreement with another study conducted in Saudi Arabia, in which 74.0% never recap the needle after administration [31].

This study showed the significant difference in knowledge and practice among urban and rural residents toward insulin injection waste disposal. The urban residents had better knowledge and practice with mean 6.81 (0.86), *p* = 0.003 and mean 8.88 (0.54), *p* = 0.001, respectively, than rural residents in Gondar town. This might be due to low contact time for counseling and absence of safe disposing option in rural area and healthcare practitioners in rural area had no good disposing practice. This finding was in agreement with a study done in Murree, Pakistan, which showed that 60% of rural health practitioners disposed of insulin injection needle at an open place [23, 32].

Respondents who join college or university had significantly higher overall mean score of knowledge and practice than those who were illiterate. This may be because they had a better chance to get information from courses and social media than those who are illiterate. However, study conducted in South Africa reported that there were no significant associations between education level and correct disposal of insulin injection waste [33]. This study revealed that there is a significant difference in knowledge and practice mean score between patients who visited and received advice from physician and who did not visit and did not receive advice frequently (who visited the physician only when getting sick) from the physician. This finding was in agreement with a study done in North East Essex, where there were statistically significant differences in practice and knowledge between patients receiving and not receiving advice on sharps disposal (AOR = 6.36 [95% CI 2.04–23.28], *p* = 0.0007) [26, 33]. This indicated that education is necessary for diabetic patients to practice good disposal of insulin injection waste. This finding was also supported by studies done in India and USA showing that education received from healthcare providers played a very important role, as shown by significant correlation [13, 26].

## 5. Limitation of Study

The study has some limitations that should be taken into account while interpreting the results. This study is conducted in only one diabetic center; the results found regarding insulin injection waste disposal may not be representative of all Ethiopian diabetic patients. As the study design is cross-sectional and depends on self-reported assessment, under- or overreporting is very likely.

## 6. Conclusion

The knowledge and practice of diabetic patients were low towards safe insulin injection waste disposal in study area. The study revealed that knowledge and practice of diabetic patients toward safe insulin injection disposing had strong association with rural residence, education level, type of DM, duration of insulin use, family history of diabetic illness, and education received from healthcare practitioners. Currently, there is no needle-disposal law or strong safe disposing system in Ethiopia provided by either local administration or ministry of health and environment. This serious issue requires urgent attention from the government to ensure the presence of safe disposal system and that the sharps are no longer disposed in the household bin or public locations like parks, buildings, or streets. Healthcare providers should also be aware of safe disposing system and counsel patients on appropriate disposal of used syringes.

## Figures and Tables

**Table 1:** Sociodemographic characteristics of respondents, Gondar, 2016.

Variable	Frequency *n* (%)
*Sex *	
Male	118 (56.2%)
Female	92 (43.8%)
*Age, in years*	
<19	6 (2.9%)
20–39	83 (39.5%)
40–59	72 (34.3%)
>60	49 (23.3%)
*Residence *	
Urban	112 (53.3%)
Rural	98 (46.7%)
*Marital status*	
Ever married	174 (82.8%)
Unmarried	36 (17.2%)
*Employment status*	
Unemployed	113 (53.8%)
Employed	97 (46.2%)
*Educational status*	
Illiterate	93 (44.2%)
Primary school	36 (17.1%)
Secondary school	50 (23.8%)
Collage/university	31 (14.8%)
*Average monthly income (in USD)*	
<75	115 (54.8%)
75–150	73 (34.9%)
>150	22 (10.5%)

**Table 2:** Disease and treatment characteristics of respondents (*n* = 210).

Variable	Frequency (%)
*Type of DM*	
Type 2	40 (19.1%)
Type 1	170 (80.9%)
*Family history of DM*	
Absent	85 (40.5%)
Present	125 (59.5%)
*Duration of insulin use*	
<1 year	44 (20.9%)
1–5 years	39 (18.6%)
>5 years	127 (60.5%)
*Daily dosing schedule for insulin*	
Once	5 (2.4%)
Twice	184 (87.6%)
*Types of insulin injection device used*	
Insulin pen	8 (3.8%)
Needle with separate syringe	42 (20%)
Insulin syringe	160 (76.2%)
More than 2 times	21 (10%)
*Source of health information *	
Physicians	153 (72.8%)
Nurses	38 (18.1%)
Others^*∗*^	
*Frequency of physician visit in the last 6 months*	
Once in a month	108 (51.4%)
Once in 3 months	82 (39%)
Once in 6 months	13 (6.2%)
Only when getting sick	7 (3.3%)

^*∗*^Pharmacists and physiotherapists.

**Table 3:** Knowledge of respondents towards insulin injection device disposal (*n* = 210).

Statement	Correct (%)	Incorrect (%)
The sharp waste produced at home is infectious	128 (61.0%)	82 (39.0%)
One can reuse needles and lancets if they are still sharp and clean	122 (58.1%)	88 (49.1%)
The needles and lancets can be cleaned by spirit swab and reused^*∗*^	109 (51.9%)	101 (48.1%)
One can also use someone else needle for injecting insulin after cleaning with spirit^*∗*^	119 (56.7%)	91 (43.3%)
Needle should be recapped after use and before throwing away in bin.	96 (45.7%)	114 (54.3%)
Needle should be broken away from syringe and collected in puncture proof bottles	88 (41.9%)	122 (58.1%)
Lancets should not be recapped after use and before throwing in waste bin	68 (32.4%)	142 (67.6%)
One should bend the lancet tip after use and before throwing in waste bin	77 (36.7%)	133 (63.3%)
Sharps like needles and lancets can cause injury if disposed in public places like parks, streets and so forth	100 (47.6%)	110 (52.4%)
The sharps in household waste can never cause injury to rag pickers and garbage handlers^*∗*^	104 (49.5%)	106 (50.5%)
Used needles and syringes can be misused by rag pickers	66 (31.4%)	144 (68.6%)
Sharps like needles can be recycled like plastics^*∗*^	199 (56.7%)	90 (42.9%)

^*∗*^Negative statement and answer false scored.

**Table 4:** Respondents' practices regarding insulin injection device waste disposal (*n* = 210).

	Frequency
*Practice score levels*	
Positive practice	40 (19.3%)
Negative practice	170 (80.7%)
*Place of insulin injection device disposal*	
Garbage	76 (36.2%)
Burned	7 (3.3%)
Buried in the ground	43 (20.5%)
Toilet	84 (40%)
*Numbers of needles thrown away in one week*	
0–7	152 (72.4%)
7–14	31 (14.8%)
>14	27 (12.9%)
*Bend the needle and sharp after use *	
No	90 (42.9%)
Yes	120 (57.1%)
*Recap the needle after injecting insulin*	
No	73 (34.8%)
Yes	137 (62.2%)
*Ask the healthcare provider about disposal of insulin syringes *	
No	166 (79%)
Yes	44 (20.1%)

**Table 5:** Mean knowledge and practice scores of respondents according to different characteristics (*n* = 210).

Variable	Total knowledge score		Total practice score	
	Mean (SD)	*p* value	Mean (SD)	*p* value
*Sex *				
Male	6.02 (0.86)	0.189	7.44 (0.59)	0.273
Female	6.54 (0.79)		7.91 (0.68)	
*Age, in years*				
<19	5.98 (0.86)	0.752	5.50 (0.63)	0.441
20–39	6.02 (0.75)		5.01 (0.64)	
40–59	5.77 (0.88)		6.33 (0.61)	
>60	5.42 (0.81)		7.44 (0.68)	
*Residence *				
Urban	6.81 (0.86)	0.003^*∗*^	8.88 (0.54)	0.001^*∗*^
Rural	4.89 (0.77)		5.45 (0.61)	
*Marital status*				
Ever married	7.21 (0.89)	0.891	6.77 (0.63)	0.386
Unmarried	6.99 (0.83)		6.12 (0.65)	
*Employment status*				
Unemployed	6.55 (0.71)	0.389	5.59 (0.67)	0.501
Employed	6.49 (0.66)		6.32 (0.84)	
*Educational status*				
Illiterate	5.23 (0.85)	0.020^*∗*^	5.22 (0.70)	0.011^*∗*^
Primary school	6.09 (0.75)		8.12 (0.53)	
Secondary school	7.44 (0.91)		8.77 (0.61)	
College/university	7.89 (0.73)		9.97 (0.78)	
*Average monthly income*				
<1000	5.99 (0.21)	0.187	6.78 (0.67)	0.300
1000–2900	6.07 (0.72)		6.45 (0.62)	
>3000	6.72 (0.74)		8.23 (0.61)	
*Type of DM*				
Type 1	8.23 (0.77)	0.000^*∗*^	9.98 (0.68)	0.005^*∗*^
Type 2	6.09 (0.81)		5.45 (0.60)	
*Family history of DM*				
Present	8.61 (0.99)	0.026^*∗*^	8.12 (0.58)	0.322
Absent	6.34 (0.97)		8.55 (0.64)	
*Duration of insulin use*				
<1 year	6.43 (0.83)	0.006^*∗*^	5.78 (0.66)	0.000^*∗*^
1–5 years	7.02 (0.69)		8.82 (0.39)	
>5 years	9.12 (0.99)		9.13 (0.14)	
*Frequency of physician visit in the last 6 months*				
Once	6.56 (0.14)	0.031^*∗*^	6.89 (0.60)	0.001^*∗*^
Twice	6.89 (0.83)		8.12 (0.68)	
More than twice	8.23 (0.91)		9.78 (0.71)	
Only when getting sick	5.76 (0.76)		4.12 (0.60)	

^*∗*^Statistically significant (*p* < 0.05).
